# Percutaneous treatment of a CTO in an anomalous right coronary artery: A rupture paved the way for new insights

**DOI:** 10.3389/fcvm.2022.916616

**Published:** 2022-07-29

**Authors:** Nino Cocco, Rosalinda Madonna, Valeria Cammalleri, Giulio Cocco, Domenico De Stefano, Danilo Ricciardi, Francesco Grigioni, Gian Paolo Ussia

**Affiliations:** ^1^Department of Cardiovascular Sciences, Campus Bio-Medico University of Rome, Rome, Italy; ^2^Cardiology Division, Department of Surgical, Medical, Molecular Pathology and Critical Care, University of Pisa, Azienda Ospedaliero Universitaria Pisana Ospedale di Cisanello, Pisa, Italy; ^3^Unit of Ultrasound in Internal Medicine, Department of Medicine and Aging Sciences, University of Chieti G D'Annunzio, Chieti, Italy

**Keywords:** anomalous right coronary artery, sudden death, anomalous aortic origin of a coronary artery, chronic total occlusion, percutaneous coronary intervention, PCI complication

## Abstract

An anomalous aortic origin of a coronary artery (AAOCA) from the opposite sinus, with an interarterial course, has been associated with an increased risk of myocardial ischemia and sudden death. As the exact pathophysiology of AAOCA is not well understood, the clinical management is also not well defined. With increased use of non-invasive imaging, the diagnosis of AAOCA is increasing and the association of anomalous origin and atherosclerotic disease is becoming a more important topic. We report a rare case of AAOCA chronic total occlusion (CTO). A 40-year-old Caucasian man was referred for invasive coronary angiography (ICA) due to typical chest pain and positive myocardial scintigraphy. ICA demonstrated CTO of an anomalous right coronary artery (ARCA) originating from the left side of the ascending aorta with an interarterial course. There was no lesion in the left coronary artery. During the procedure, unexpected rupture of the coronary artery occurred after dilatation with a small balloon at low pressure. The complication in this case was handled with good procedural final result but was an occasion for a food for thought. Coronary artery perforations are rare but life-threatening procedural complications that are usually caused by predisposing anatomical and procedural factors. We issue a warning on the risk of complications during complex percutaneous coronary intervention of these arteries, and we reconsidered the pathophysiology of the anomaly in a way that could change the approach to the disease. Based on this complication, we hypothesized that the wall of the artery could be fragile due to histopathological alterations, which could have a role in the pathophysiology of coronary malignancy. Future autopsy studies should be focused on the analysis of the arterial wall of the patient affected by sudden death with this anomaly.

## Introduction

An anomalous aortic origin of a coronary artery (AAOCA) from the opposite sinus, with an interarterial course between the pulmonary artery and aorta, has been associated with an increased risk of myocardial ischemia and sudden cardiac death (SCD) (1–4). The degree of risk (5, 6) and the pathophysiology of myocardial ischemia (7, 8) are debatable. Guidelines are limited and recommendations are mainly based on expert opinions (9, 10). Moreover, with increased use of non-invasive imaging to evaluate coronary artery disease, an increase in the absolute numbers of AAOCA is expected, with a concomitant increase in the prevalence of coronary artery disease. The management of patients affected by anomalous origin and atherosclerotic disease is becoming a more important topic in the field of coronary artery disease (11). We report a rare case of an anomalous right coronary artery (ARCA) originating from the left side of the ascending aorta with symptomatic chronic total occlusion (CTO). An unexpected complication led us to reconsider the pathophysiological basis of ARCA and to suspect histological involvement of the arterial wall.

The goals of this paper are three-fold: (1) to attempt an estimation of SCD specific for right AAOCA (ARCA) based on extrapolations of incidence numbers of SCD in the population and report the prevalence of right AAOCA, (2) to review the presumed pathophysiology of SCD in ARCA and present new insights, and (3) to issue a warning on the risk of complications during percutaneous coronary intervention (PCI) for ARCA CTO.

## Case description

**Figure 1:**
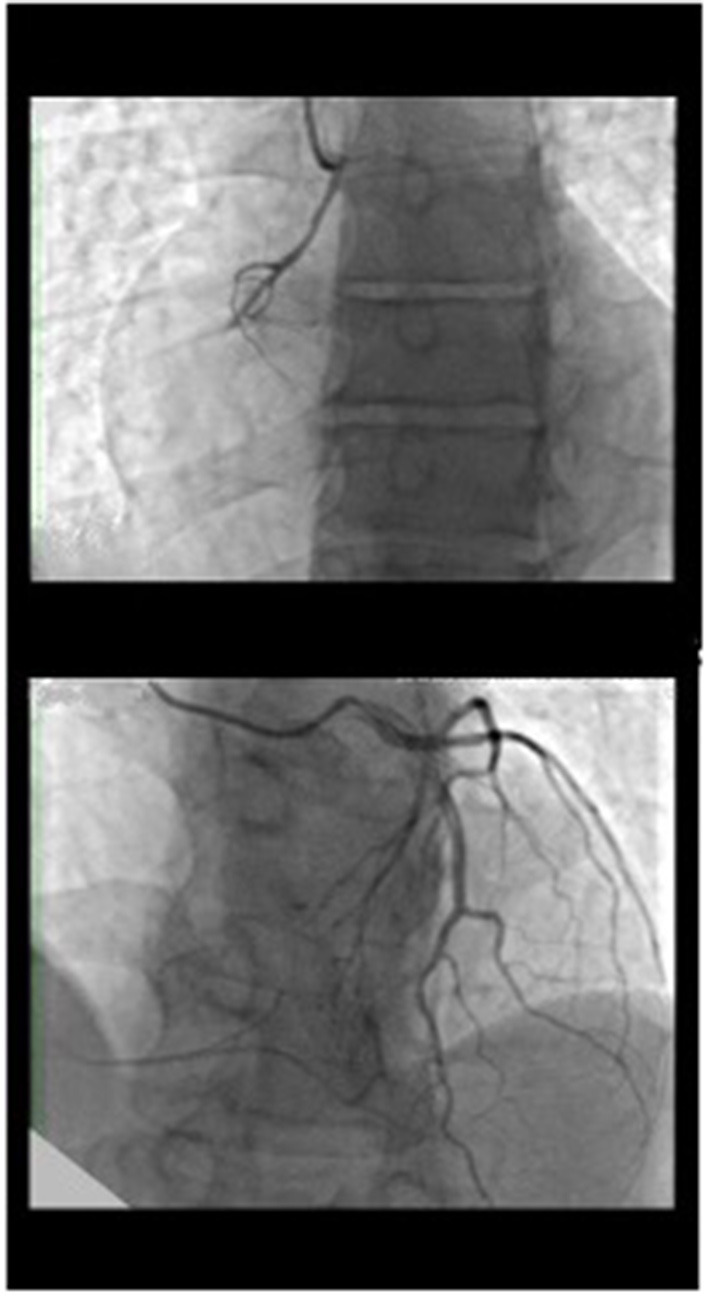
Coronary angiography of the left system showed left coronary artery normally positioned without atherosclerotic disease with rentrop III heterocoronary collateral circulation toward the right coronary artery, which has an anomalous origin on the left side of the ascending aorta and chronic occlusion in the proximal atrioventricular tract.

**Figure 2:**
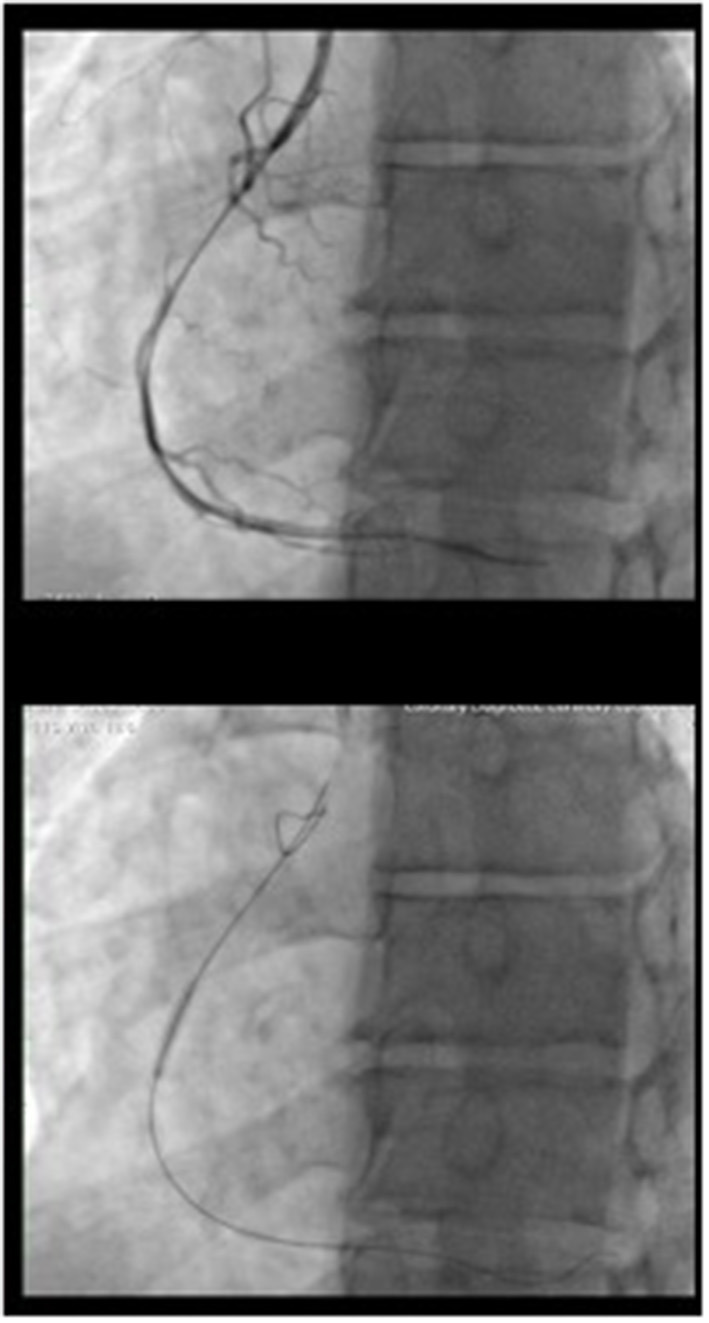
CTO treatment procedure: The occlusion was crossed with a tapered polymeric guidewire supported by microcatheter. The flow was restored after a dilatation of the occlusion with 1.5 x 12 balloon. Than a dilatation with a 2 x 15 balloon at 10 atm was performed.

**Figure 3:**
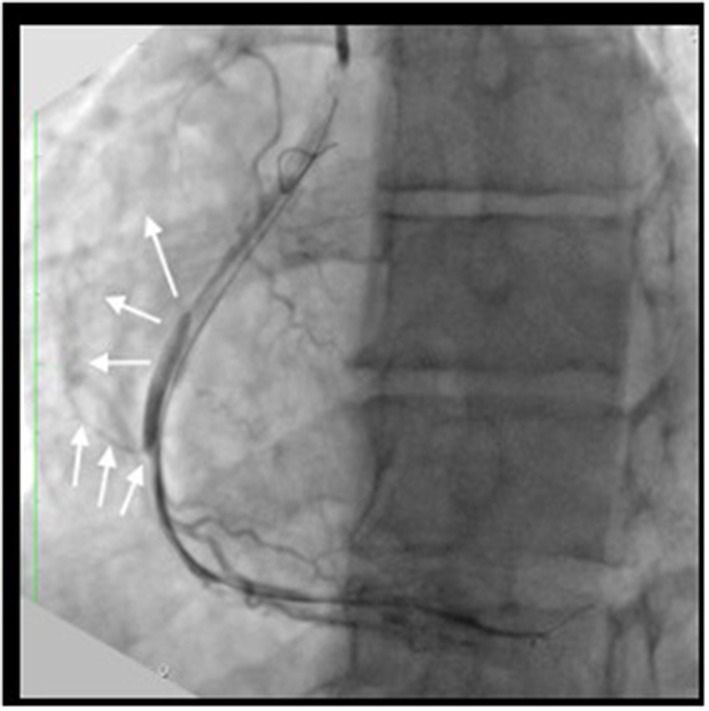
Procedural complication: The angiography after CTO dilatation with compliant 2 x 15 mm balloon at 10 atm showed coronary Ellis II perforation (white arrow) and diffuse type D dissection.

**Figure 4:**
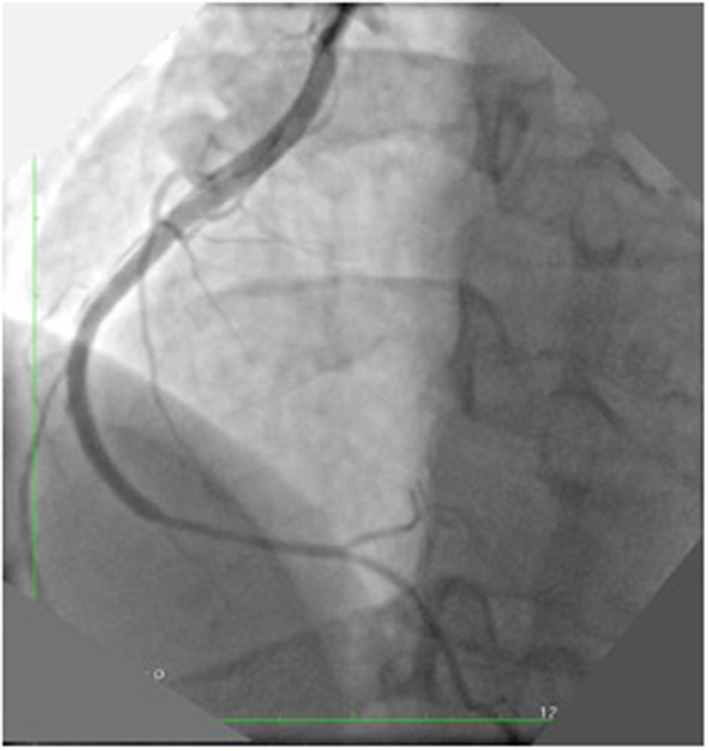
Post PCI final result: A first stent was implanted in the ruptured tract with long inflation (5 min). After the deployment the effusion disappeared. Four additional stents were implanted to cover the dissection. The final angiography showed a good result.

**Figure 5:**
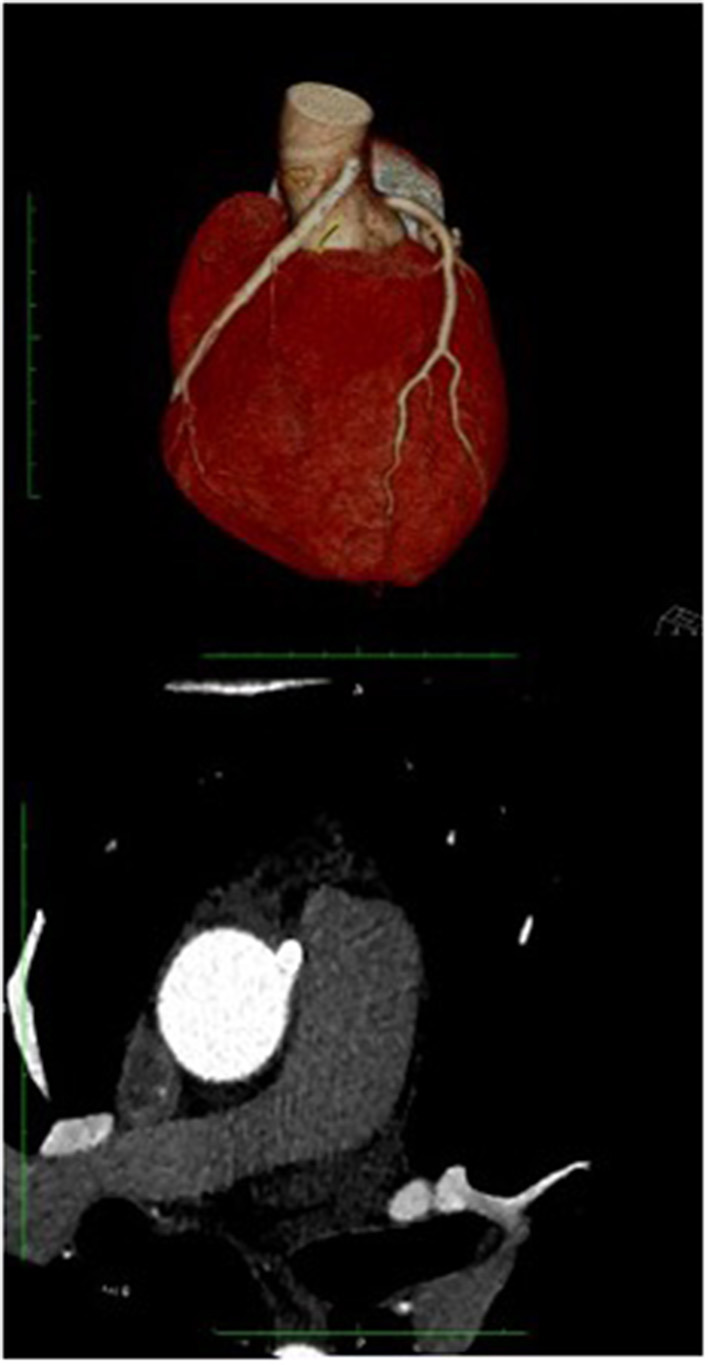
Coronary TC: The coronary TC confirmed the anomalous origin from opposite site of ascending aorta 17 mm from the sinotubular junction, with interarterial course. Modest effusion (10 mm).

A 40-year-old Caucasian man, without any remarkable risk factors, was hospitalized for investigation and treatment of a several-month-history of class II angina. Myocardial scintigraphy, three weeks prior, showed moderate reversible perfusion defects involving the inferoposterior wall area. The patient was previously healthy and participated in non-competitive soccer. A 12-lead electrocardiogram and baseline echocardiography showed no abnormal wall motion and well-functioning valves. A baseline angiogram was performed from the right radial artery. The left coronary artery, originating from the left sinus, did not exhibit significant stenosis. There was Rentrop III collaterality towards the right coronary artery (RCA). The RCA, originating high and anteriorly over the left sinus, showed CTO in the middle tract (Figure 1). A Jr4 guide-catheter was chosen and a 1,5 × 8 mm anchoring balloon was placed in the conus artery. The occlusion was smoothly crossed with a tapered polymeric guidewire supported by a 1.8 Fr microcatheter. The attempt to bring the microcatheter beyond the occlusion failed because of the friction encountered, so we performed a predilatation with 1.5 × 12 mm compliant balloon. Good anterograde flow was restored (Figure 2). A second dilatation with a 2 × 15 mm balloon at 10 atm was performed to better prepare the lesion. A few seconds after this dilatation, the patient experienced chest pain. Angiography revealed a type II Ellis perforation in the midline and an extensive type D dissection distally to the ostium (Figure 3; Supplementary Video 1). A 3 × 26 mm zotarolimus eluting stent was implanted with long inflation (5 min). After deployment, the effusion disappeared. Four additional stents were implanted to cover the dissection. The final angiography showed a good result (Figure 4; Supplementary Video 2). Echocardiography showed negligible pericardial effusion. The patient was clinically stable and asymptomatic. Troponin levels remained stable on two occasions. A coronary computed tomography scan (CTA) confirmed the anomalous antero-left high origin of the RCA 17 mm from the sinotubular junction, and showed the angled ostium and interarterial course, about 2 cm between the aorta and pulmonary trunk (Figure 5), with modest effusion (10 mm) on CTA.

## Discussion

### Prevalence of ARCA and risk of sudden death

Coronary artery anomalies have been reported as the second most frequent cause of SCD in young athletes, accounting for 12% of deaths (12, 13). Coronary artery anomalies arising from the pulmonary artery or the wrong sinus with an interarterial course between the pulmonary artery and aorta, referred to as AAOCA, are more often related to sudden death (SD) (14, 15). The most common subtype is an ARCA arising from the left side (prevalence 0.23%), followed by an anomalous left coronary artery (ALCA) arising from the right side (prevalence 0.03%) (1, 16). ALCA from the pulmonary artery is extremely rare (11, 15). Although evidence demonstrates that interarterial ALCA and ARCA are associated with an increased risk of SCD, their absolute risk in the general population is unknown. Thus, controversy remains regarding the optimal approach to risk stratify and manage these patients. With increased use of non-invasive imaging, the prevalence is increasing (1, 16) and could become a serious public health hazard.

The first data on the rates of SCD in AAOCA came almost exclusively from autopsy series, which reported high, yet discrepant, mortality rates between studies (0-50% for ARCA and 30-100% for ALCA) (1, 4, 12). In the first series, 50% of SD cases had anomalous coronary arteries (30% right and 80% left) (1, 4, 12). From this data, the estimated risk of SCD in AAOCA was very high (50% for ALCA and 25% for ARCA) (17, 18) and many authors agreed that surgical correction should have been the first line of therapy in these patients (19–22). However, this autopsy statistic report has been proven wrong. It reported on the chance of a person who dies suddenly having an anomalous coronary artery, not the risk of SD from an anomalous coronary artery, only reflecting the prevalence of AAOCA in those who died. Subsequent studies indicated that the risk of SD was far lower, but discrepancy in the data remains. The rate of death from AAOCA has been reported as being between 0.07 and 0.61 per 100,000 person years (5, 12, 16, 20, 22). From this data, it was determined that the cumulative risk of death over a 20-year period from the age of 15–34 years in patients with AAOCA was 6.3% for ALCA and 0.2% for ARCA (23). Assuming the prevalence of AAOCA is 0.1-0.2% (24–27), the risk of death for AAOCA is 0.12–0.15% (1:860–1:652), involving above all young people engaged in frequent vigorous exercise and patients with ALCA (5, 28). A more recent review of a database of 5,100 cases of SCD referred to a specialist cardiac pathology center between January 1994 and March 2017, and identified a subgroup of 30 cases (0.6%) with AAOCA (15). The number of SCD from ARCA and ALCA were compared, and, although ALCA is considered more malignant, the SCD numbers for ARCA were slightly higher (11 patients, 37% vs. 10 patients 37%, respectively). ALCA from the pulmonary artery occurred in seven cases (23%). Exercise-induced SCD was associated more frequently with ALCA than ARCA, where death occurred often at rest or during sleep. The mean age was much higher, compared to previous studies (28+/−17 years); there were patients in the fifth and sixth decades of life, most with ARCA.

The risk of SCD in patients with AAOCA are inconclusive due to the conflicting results. We believe this discrepancy could have stemmed from the fact that ARCA and ALCA have different clinical features and the identification of a unique risk of AAOCA (29) could have been the original reason for the conflicting results in the literature. SCD was more likely to occur in ALCA than ARCA; in fact, SCD in young, trained patients are mainly caused by ALCA. But, when we consider the populations of each age group, the number of SCD cases were equivalent or even greater for ARCA (15), since ARCA was more prevalent (0.23 vs. 0.03%) (6, 11, 30). In patients of older age, death often occurs at rest or during sleep (15). Thus, ARCA is not a minor disease but a different disease, most likely with a different pathophysiological pathway as well. The risk of SD in a patient with ARCA has not been properly analyzed. We attempted to estimate the risk of SD based on the data from previous studies.

The age-adjusted national incidence of SCD is 60 per 100,000 (95% confidence interval; 54–66 per 100,000) (31, 32) and 0.6% thereof are caused by AAOCA (15), translating to an incidence of 3,6 per 1,000,000 per year. ARCA are found in 36% of SCD caused by AAOCA (15), translating to an incidence of 1,3 per 1,000,000 per year. Since the prevalence of ARCA is 0.23%, we can derive that 1,3 per 2300 persons a year with ARCA experience SCD. Therefore, the incidence of SD in a patient with ARCA is about 1:1800. This is the first attempt to estimate the incidence of SCD in a patient specifically affected by ARCA.

### Anomalous origin of a coronary artery from the opposite sinus pathophysiology: The enigma of the true denominator

Assuming the pathophysiological understanding of the disease is key to predict the risk of SCD in AAOCA; several studies have attempted to isolate a key primary factor, but no single anatomical component of the coronary artery has been isolated. Several studies have been done to find a true “denominator” causing ischemia and SCD. Several putative mechanisms have been proposed to account for the association between SCD and an anomalous origin of a coronary artery from the opposite sinus. The downstream event is presumed to be ischemia (8, 33–37). Historically, the interarterial course was thought to be the crucial abnormality, assuming a scissor-like mechanism created by the close proximity of the aorta and pulmonary artery, especially during exertion (1, 2, 8). However, the pulmonary artery is unlikely to exert sufficient pressure to occlude a coronary artery in the absence of pulmonary hypertension. Therefore, the interarterial course may act only as a surrogate for other anatomical high-risk features, including a slit-like ostium, acute take off angle, and proximal narrowing (also referred to as hypoplasia) with an elliptical vessel shape and intramural course. The anatomical high-risk feature of a slit-like ostium at the ectopic origin is defined as a ≥50% reduction in the minimal lumen diameter compared to the normal distal reference diameter (34). Thus, the deformed coronary ostium with a decreased cross-sectional area acts as an ostial stenosis. Due to the slit-like ostium opening, during exercise and systolic expansion of the aortic root, the orifice can collapse in a valve-like manner (34, 35). An acute take-off angle (less than 45°) is defined as an axial course of the proximal segment tangential to the great vessel circumference. Kinking of an anomalous coronary artery during exercise was proposed as a contributing ischemia-inducing mechanism (8, 34). The intramural course length is considered the most relevant feature in terms of hemodynamic repercussion (6, 35–37). The intramural coronary artery is slit-like and is characterized by thin inner and outer aortic wall layers and lateral luminal compression that undergoes phasic increases with each systole (38). This anatomical feature is considered by many authors as being related to SCD, especially if associated with the other features mentioned above (37). Unfortunately, in clinical practice, none of these have been shown to be useful in the prognostic assessment of SD and there are no prognostically distinguishing pathological features that could be prospectively defined (7–10). Finally, another theory postulates that compression and kinking of the anomalous artery could cause intimal damage and a propensity to spasm and ischemia (39, 40), which was described more than 20 years ago, but considered implausible by the majority (8, 41).

We describe a rare case of anterograde CTO of the RCA with anomalous origin from the opposite side of the ascending aorta. Data regarding complex PCI, especially for CTO, in this setting are very limited (24, 26, 27, 42–48). The procedure was unexpectedly complicated by perforation and extensive type D dissection after predilatation with a small balloon at low pressure. Coronary perforation is a rare, but life-threatening, complication. It is usually due to high pressure balloon dilatation or the use of an oversized balloon in the context of predisposing factors such as tortuosity, calcification, old age, CTO, or small vessel caliber (24). The myocardial bridge is also an important predisposing factor (25). A 2 mm diameter balloon inflated at 10 atm in a 3.5 mm diameter artery causing perforation is almost inexplicable, even in a CTO procedure. The rupture could be explained by structural fragility of the artery. An anomalous course with acute angulation may favor the complication but there could also be a histopathological explanation. In the myocardial bridge, for example, the anomalous course of the artery is associated with intimal and endothelial differences in the tunneled artery that predispose to a greater risk of rupture during PCI. The intimal layer is thinner, and the endothelial cells are spindle shaped. This alteration and compression carry a major risk for perforation (25). Bunji et al. (39) hypothesized that the RCA located between the aorta and the pulmonary artery is prone to compression or kinking phenomena, resulting in intimal disruption and subsequent predisposition to vasospasm that correlates with ischemia and SCD. The histopathological alteration causing wall fragility could also be an explanation for the complication in our case.

PCI in an anomalous coronary artery is difficult, particularly with CTO, due to technical complexities, from engaging the coronary ostium to delivery of hardware through the vessel. Only a few cases have been reported in the literature (24, 26, 27, 42–48) (Table 1).

**Table 1:** ARCA CTO procedures reported in the literature.

**Cases**	**Age/sex**	**Technique used**	**Origin of the RCA**	**Site of occlusion**	**Complications**
Kaneda et al. (45)	66 y/M	Antegrade	Left sinus	Proximal atrioventricular tract	None
Fang et al. (47)	74 y/M	Antegrade	High anterior	Proximal atrioventricular tract	Cusp dissection
Abdou and Wu (27)	58 y/M	Antegrade	Left sinus	Proximal atrioventricular tract	Aorto-coronary dissection
Porwal et al. (44)	71 y/F	Antegrade	Left sinus	Proximal atrioventricular tract	None
Senguttuvan et al. (43)	49 y/M	Antegrade	Left sinus	Proximal atrioventricular tract	None
Gasparini et al. (46)	65 y/M	Retrograde	Left sinus	Proximal atrioventricular tract	None
Yamada et al. (42)	43 y/M	Retrograde	Left sinus	Ostial	None
Young et al. (48)	68 y/M	Antegrade	Left sinus	Proximal atrioventricular tract	None
Patra et al. (26)	58 y/M	Antegrade	Left sinus	Proximal atrioventricular tract	None
Our case	40 y/M	Antegrade	High anterior left aorta	Proximal atrioventricular tract	Dissection and perforation

**Figure 6:**
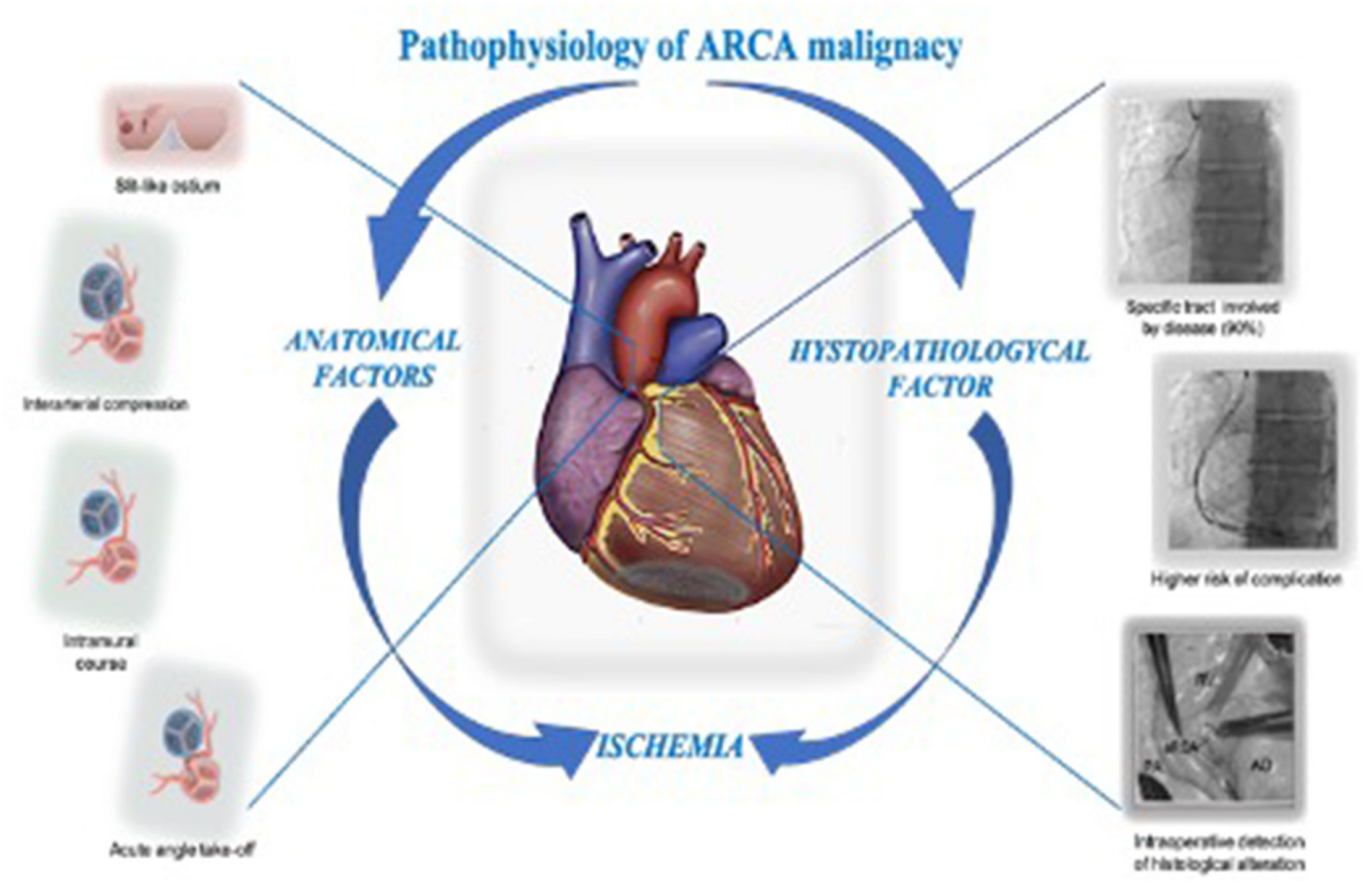
ARCA presumed ischemia pathophysiology: The downstream event in the pathophysiology of ARCA malignancy in the contest of SCD is presumed to be ischemic. The anatomical feature like the interarterial passage causing compression in a scissor-like mechanism, the slit-like ostium acting as an ostial stenosis, the acute take-off angle causing kinking, and the intramural course causing compression, are historically considered involved in the pathogenesis of ischemia. Unfortunately, in clinical practice none of these have been shown to be useful in the prognostic assessment of SD. The higher incidence of procedural complications in the context of ARCA CTO treatment, the presence of a specific tract of the artery involved by the disease (90% of the case reported in literature), and the intraoperative detection of histological alteration of the anomalous artery wall in a patient who suffered from a SCA call us to reconsider the histopathological alteration in the pathogenesis of ischemia and ARCA malignancy. ARCA, anomalous right anomalous coronary originating from the left side; CTO, chronic total occlusion; SCD, sudden cardiac death.

Nine cases of ARCA CTO have been reported in the literature. Three major complications, two of which were not related to catheter manipulation, were mentioned. A case of aorto-coronary dissection due to balloon dilatation and rupture was described by Abdou and Wu (27). In this case, the type D dissection started from the proximal ARCA and extended retrograde, involving the aorta. Estimation of the risk of complication is not the purpose of this paper, considering the small number of patients, but this trend is unusual. Furthermore, analysis of the cases showed a common tract of occlusion in most cases. Nine out of ten cases involved the proximal atrioventricular part of the artery, in the area between the cone branch and the first branch for the acute margin (27, 42–48). Due to the difference in anatomy of the anomalous artery, we divided it into four segments. We considered the proximal segment to be the tract from the origin to the origin of the conus or sinoatrial branches, the proximal atrioventricular segment the tract from the conus branch to halfway to the acute margin, the middle segment the tract from this halfway point to the acute margin, and a distal segment from the acute margin to the base of the heart at the junction of the atrial and ventricular septum. Analyzing the non-CTO PCI cases in the literature, we confirmed that the tract of disease in ARCA was almost always the proximal atrioventricular segment. A review of 18 cases with imaging showed that 16 had proximal atrioventricular segment involvement (49–62), five of six had SCA- STEMI (58–62), and 11 of 12 had chronic critical stenosis (49–58). Furthermore, Bruls et al. (63) described a case of resuscitated SD in a 37-year-old man with ARCA in whom they performed coronary artery bypass grafting. After surgical examination of the artery, several initial branches in its first segment (infundibular branch or the sinus nodal artery) and a fibrously thickened wall were noted. These data suggest that histological alteration may play an important role in ARCA malignancy (Figure 6).

There is no greater tragedy in medicine than SD in an apparently healthy person. AAOCA is the second leading cause of SCD in athletes, and with increased use of non-invasive imaging, the prevalence is expected to increase. The interarterial course is believed to be the factor of malignancy in this context and a lot of effort has been made to find a true denominator in understanding the pathophysiological pathway. After almost 50 years, none of the suspected anatomical factors have been shown to be useful in the prognostic assessment of SD and there are no prognostically distinguishing pathological features that could be prospectively defined. Therefore, the clinical management is still not defined. The strategy of the common pathway causing malignancy has failed, thus, the interarterial course may act only as a surrogate. ARCA and ALCA have very different clinical features. ALCA is rare but more malignant and, among AAOCA patients, causes most SD cases in young patients. ARCA is relatively common and less malignant, but when populations of each age group are evaluated, the number of SCDs it causes are equal, or even greater than ALCA. These patients are older, and deaths often occur at rest. Therefore, ARCA is not a minor disease, but a different disease. Based on this assumption, the pathophysiological basis of malignancy could also be different. In our case, an unexpected complication led to reconsideration of a possible parietal structural involvement in ARCA malignancy. The theory of histological alteration of the arterial wall playing a role in the pathophysiology of ARCA malignancy is plausible based on three observations: (1) the higher incidence of coronary periprocedural major dissection; (2) the evidence of a specific tract of the artery being affected by disease (90% of the cases reported), which may render it particularly prone to stress, torsion, or kinking, and thus more prone to histological alteration; and (3) the intraoperative detection of wall alteration of the anomalous artery that was demonstrated during surgical examination of the first tract of ARCA in a 36-year-old patient.

The istologyical alteration causing spasm and ischemia, suspected by some authors but considered implausible by the community, especially after studies performed with intracoronary acetylcholine, would seem to disprove this hypothesis. We hypothesized that this alteration could involve ARCA in specific segments and that spasm may not be the only factor for ischemia. This alteration could render arteries more prone to compression, kinking, atherosclerosis, or thrombus. We also hypothesized that each patient may have a different involvement and that the few patients with more important histological distortion could be those at most risk. The histological pattern of the anomalous arterial wall was never evaluated.

In conclusion, 50 years since the identification of the relationship between SCD and AAOCA, the risk of SCD in a carrier patient has still not been defined and the pathophysiological causes of the disease are unclear. For the first time in the literature, we issue a warning on the risk of complications during percutaneous treatment of a CTO in an anomalous right coronary artery and we reconsidered the pathophysiological basis of the anomaly in a way that could change the approach to the disease.

## Author contributions

All authors listed have made a substantial, direct, and intellectual contribution to the work and approved it for publication.

## Conflict of interest

The authors declare that the research was conducted in the absence of any commercial or financial relationships that could be construed as a potential conflict of interest.

## Publisher's note

All claims expressed in this article are solely those of the authors and do not necessarily represent those of their affiliated organizations, or those of the publisher, the editors and the reviewers. Any product that may be evaluated in this article, or claim that may be made by its manufacturer, is not guaranteed or endorsed by the publisher.

## Abbreviations

AAOCA: Anomalous aortic origin of a coronary artery
ALCA: Anomalous left coronary artery
ARCA: Anomalous right coronary artery
CTO: Chronic total occlusion
ICA: Invasive coronary angiography
PCI: Percutaneous coronary intervention
RCA: Right coronary artery
SD: Sudden death
SCD: Sudden cardiac death.
